# Exercise strategies to protect against the impact of short-term reduced physical activity on muscle function and markers of health in older men: study protocol for a randomised controlled trial

**DOI:** 10.1186/s13063-016-1440-z

**Published:** 2016-08-02

**Authors:** Oliver J. Perkin, Rebecca L. Travers, Javier T. Gonzalez, James E. Turner, Fiona Gillison, Cassie Wilson, Polly M. McGuigan, Dylan Thompson, Keith A. Stokes

**Affiliations:** 1 Department for Health, University of Bath, Claverton Down, Bath, BA2 7AY UK; 2 Arthritis Research UK Centre for Sport, Exercise and Osteoarthritis, Nottingham, UK

**Keywords:** Reduced activity, Step-reduction, Muscle, Strength, Atrophy, Inactivity, Resistance exercise, Recovery

## Abstract

**Background:**

Muscles get smaller and weaker as we age and become more vulnerable to atrophy when physical activity is reduced or removed. This research is designed to investigate the potentially protective effects of two separate exercise strategies against loss in skeletal muscle function and size, and other key indices of health, following 14 days of reduced physical activity in older men.

**Methods:**

Three groups of 10 older men (aged 65–80 years) will undertake 2 weeks of reduced activity by decreasing daily steps from more than 3500 to less than 1500 (using pedometers to record step count). Two of the three groups will then undertake additional exercise interventions, either: 4 weeks of progressive resistance training prior to the step-reduction intervention (PT-group), or home-based ‘exercise snacking’ three times per day during the step-reduction intervention (ES-group). The third group undertaking only the step-reduction intervention (control) will provide a comparison against which to assess the effectiveness of the protective exercise strategies. Pre and post step-reduction assessments of muscle function, standing balance, anthropometry and muscle architecture will be taken. Pre and post step-reduction in postprandial metabolic control, resting systemic inflammation, adipose inflammation, oxidative stress, immune function, sleep quality, dietary habits, and quality of life will be measured. The stress response to exercise, and signalling protein and gene expression for muscle protein synthesis and breakdown following an acute bout of exercise will also be assessed pre and post step-reduction. Rates of muscle protein synthesis and adipose triglyceride turnover during the step-reduction intervention will be measured using stable isotope methodology. All participants will then undertake 2 weeks of supervised resistance training with the aim of regaining any deficit from baseline in muscle function and size.

**Discussion:**

This study aims to identify exercise strategies that could be implemented to protect against loss of muscle power during 2 weeks of reduced activity in older men, and to improve understanding of the way in which a short-term reduction in physical activity impacts upon muscle function and health.

**Trial registration:**

ClinicalTrials.gov: NCT02495727 (Initial registration: 25 June 2015)

## Background

The ageing process is accompanied by a progressive loss of muscle size and strength, and data from longitudinal studies suggests that muscle size is lost at 0.5–1 % per year after the age of 50 [1]. With ageing, muscle strength is lost two to five times more rapidly than would be expected based on the rate of muscle mass loss [2], and at twice the rate in the legs compared to the upper body [3]. Muscle power is the product of force and velocity of movement, and it is evident that power decreases with age to an even greater extent than strength [4]. Power provides an indication of the neuromuscular component of muscle function and thus is considered to be a better predictor of functional status than muscle mass or strength alone [5, 6]. Frailty is largely caused by this loss of muscle function [7, 8], eventually resulting in the tasks of daily living becoming too physically strenuous to be managed safely, and independent living becoming untenable.

Physical activity has been shown to be vitally important for maintaining muscle mass and function. During periods of dramatically reduced activity, such as bed rest, the muscles of healthy individuals rapidly lose size, strength and power, most notably from the legs [9, 10], with older individuals seemingly more vulnerable to this complete ‘disuse-induced’ muscle atrophy than younger individuals [11, 12]. In a population of generally healthy older individuals, however, extended periods of bed rest are unlikely to occur unless accompanied by severe injury or illness that may cause muscle wasting independently. Periods of reduced activity in healthy older adults are more likely to be the result of minor injury or illness, or extended bad weather [13, 14]. Scenarios such as these would result in a reduction in the number of steps walked on a daily basis for a short period of time, rather than total bed rest. The only previous investigation to focus on this step-reduction model of reduced physical activity in older people without any protective interventions demonstrated that reducing walking to less than 1500 steps per day for 2 weeks resulted in an average loss of around 4 % of leg muscle tissue, but with some participants losing up to 9 % [15]. In light of the estimated annual loss of 0.5–1 % of muscle size this much larger loss due to step-reduction is a concerning observation, particularly with periods of reduced activity occurring more often in older age [13]. However, in the aforementioned study, muscle strength was not reduced by the period of step-reduction, which is surprising given that strength is typically lost more rapidly than muscle size [16]. It should be noted that muscle strength was measured by assessing unilateral isometric knee extensor torque using a dynamometer for which extensive familiarisation has been demonstrated to be crucial to obtain reliable results [17]. Furthermore, changes in muscle power cannot be inferred from isometric dynamometry. Given that the nervous system is shown to adapt to changes in physical activity levels more rapidly than the contractile structures of muscle tissue [18], muscle power may be more sensitive to periods of reduced activity than maximum-force-producing capabilities. An additional and critically important role of the neuromuscular system is to maintain postural balance. If the neuromuscular system is compromised after a period of reduced activity it could be hypothesised that balance would be affected leading to an increased risk of falling [19, 20].

With only two studies previously investigating this topic specifically in an older population [15, 21], many questions remain as to the precise physiological mechanisms causing such marked response in muscle tissue to a seemingly benign and common scenario [22]. Although there is a wealth of literature highlighting the importance of maintaining physical activity for many elements of healthy ageing [23], relatively little direct evidence exists regarding the impact of acutely reducing activity in already healthy individuals. The physiological responses occurring when physical activity levels change are not confined to single tissues or organs [24], and it is crucial to understand the interactions between tissues that result in negative health outcomes such as muscle loss, particularly in an older population. Gaining an understanding of the impact of short-term reductions in physical activity is also a crucial precursory step to identifying protective interventions with real-world applicability. Resistance exercise is widely regarded to be the primary countermeasure against ‘disuse-induced’ muscle wasting [10, 25, 26]. In circumstances in which a period of reduced activity is scheduled but physical activity will subsequently be limited, for example, following to minor elective surgery, physical preparation through a concentrated period of resistance training may raise the ‘baseline’ muscle function and health profile such that reduced activity-induced muscle loss is compensated for. This may present a strategy to improve recovery from reduced activity as performance tasks of daily living should be no more challenging than prior to the pre-training, thus potentially allowing return to previous habitual activity levels more quickly.

There may of course be circumstances in which reduced activity is unavoidable but capacity to exercise is not compromised, for example, during extended inclement weather. Devries et al. [21] demonstrated that laboratory-based low-load resistance thrice weekly in fact increased leg lean tissue in older men during 2 weeks of step-reduction to less than 1500 steps/day. However, restricted access to resistance exercise equipment may render this exercise strategy infeasible; thus, home-based, body weight exercise may be a practical alternative to low-load resistance training. Previously, other health indices (e.g. metabolic health and mobility in frail older people) have been demonstrated to be improved by such exercise regimes [27, 28]. Again, this presents an opportunity to prevent decline in muscle function due to periods of reduced physical activity that may contribute to frailty, increased risk of falls, and their associated negative implications.

### Study objectives

The primary aim is to determine the protective effects of two exercise interventions against the loss of lower limb skeletal muscle power in healthy men aged 65–80 years undertaking 14 days of reduced physical activity (less than 1500 steps per day). The two exercise interventions are either: (1) undertaking 4 weeks of resistance training prior to the 14 days of step-reduction, or (2) home-based ‘exercise snacking’ during the 14 days of step-reduction. Alongside this, the influence of the step-reduction intervention and potential protective effects of the exercise interventions on other indices of muscle function (strength and neural drive), standing balance, anthropometric measures, metabolic health, rate of muscle protein and adipose triglyceride synthesis during ‘free-living’, markers of inflammation and oxidative stress at rest and in response to exercise, immune function (e.g. lymphocyte cytokine production), dietary habits, sleep quality and quality of life will be assessed. Thereafter, the possible benefits of the exercise interventions on recovery of muscle function and size will be investigated. Once all elements of the study are completed, the acceptability of the interventions and feasibility of adhering to the approaches of the recommended exercise strategies will be assessed, and user-generated input for improvements to future follow-on work obtained.

### Study design

The methods described herein have been approved by the National Health Service (NHS) South West Cornwall and Plymouth Research Ethics Committee (REC) with an allocated REC reference number: 15/SW/0130. The project was subsequently registered as a clinical trial (ClinicalTrials.gov: NCT02495727). The Standard Protocol Items: Recommendations for Interventional Trials (SPIRIT) checklist was completed.

The study will address the objectives by way of a three-group randomised control trial. One group will act as a ‘control’ against which to compare the potential protective effects of exercise interventions insofar as this group will undertake step-reduction without either exercise intervention. Hypotheses: (1) reduced activity alone will result in loss of leg muscle power, (2) exercise pre-training will increase leg muscle power above baseline so any loss of muscle power due to the reduced activity period will be offset to such an extent that muscle power will remain above pre-training baseline, and (3) daily exercise snacking during the reduced activity period will attenuate the decline in leg muscle power due to reduced activity.

## Methods

### Recruitment

The study will be advertised on the University of Bath web pages, and on posters placed around the University of Bath campus and distributed to local community clubs for older people in the Bath area. A press release will be sent to local newspapers and radio and television media outlining the study objectives and it is expected that interviews will be carried out as a result to raise awareness of the study.

### Inclusion criteria

In order to participate in this study, volunteers must satisfy all of the following criteria:

- Be aged 65 to 80 years
- Not be underweight or obese (body mass index (BMI) ≥20 and ≤30 kg/m^2^)
- Be a non-smoker (for more than 5 years)

### Exclusion criteria

Participants will not be able to take part in this study if they meet any one of the following criteria:

- Have any chronic illness, cardiac, pulmonary, liver, or kidney abnormalities, uncontrolled hypertension, peripheral arterial disease, insulin- or non-insulin dependent diabetes or other metabolic disorders
- Are individuals who consume on a daily basis any analgesic or anti-inflammatory drug(s), prescription or non-prescription
- Are individuals taking medications known to affect protein metabolism (i.e. corticosteroids, non-steroidal anti-inflammatory drugs, or prescription-strength acne medications)
- Are individuals with a history of bone, joint or neuromuscular problems or a current musculoskeletal injury
- Are individuals with a known bleeding disorder, take the anticoagulant drug warfarin, or are prone to keloid scarring
- Are individuals with a known negative reaction to the local anaesthetic lidocaine
- Are individuals with any joint replacement surgical implants or other artefacts containing metal
- Are individuals who completed fewer than 3500 steps per day (as assessed by pedometer) prior to the study
- Are individuals who score less than 8 on the Short Physical Performance Battery [29]

### Population and sample size

Thirty healthy men (non-smokers without diabetes) aged 65–80 years will be recruited and randomised into three groups of 10 by way of minimisation to limit between group differences in mean age, BMI and habitual steps/day. Participant and researcher blinding to group allocation is not possible, and screening and group allocation will be implement by the same researcher for all participants

The study is powered to detect the protective effect of the aforementioned exercise strategies against loss of leg muscle power during 14 days of reduced activity. Over 14 days of step-reduction a mean (SD) decrease of 3.9 (2.4 %) in lower limb muscle mass is anticipated [15], with measures of muscle power expected to decline at a similar or faster rate; thus, seven participants per group (95 % power and 5 % alpha) will be required to observe changes in leg muscle strength. The decline in muscle power is expected to be the same in the pre-training group as in the control group, hence significant increases in leg muscle power prior to reducing physical activity are required. Significant increases in leg strength in older men with 4 weeks of pre-training could be observed using nine participants (95 % power and 5 % alpha) [30], and it expected that this would be equivalent in muscle power. It is difficult to predict the extent of the protective effect likely to be provided by the exercise snacking. Therefore, the sample size will be rounded to 10 participants per group to account for drop out and the potentially more subtle changes that might be observed in the secondary measures.

### Overview of study procedures

**Fig. 1:**
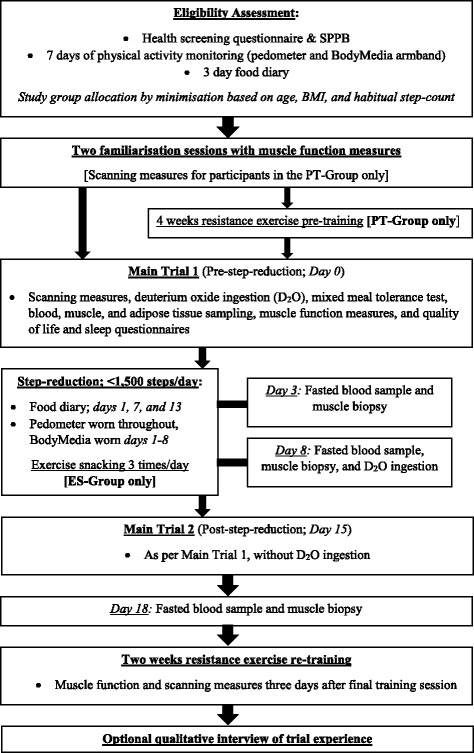
A flow diagram of the study timeline. SPPB, Short Physical Performance Battery; BMI, bodymass index; PT-Group, pre-training group; ES-Group, exercise snacking group

See Fig. 1 for a flow diagram of the study protocol. Following baseline assessment of habitual steps/day, to ensure familiarity with functional measures, all participants will attend two familiarisation sessions. Participants in the step-reduction intervention (PT-group) will then undertake 4 weeks of resistance exercise training. All participants will undertake a main trial the day before commencing 14 days of reduced activity by step-reduction to less than 1500 steps per day. On days 3 and 8 of the step-reduction protocol, a resting muscle biopsy sample will be taken. The day after the step-reduction period, a follow-up main trial will be conducted as per the first main trial, followed 3 days later by a final resting muscle biopsy. All participants will undertake 2 weeks of laboratory-based resistance re-training and a final assessment of leg muscle function and anthropometry, with the option of a follow-up qualitative interview of their experience of the trial. Thereafter, all participants will be offered a 3-month membership to the University of Bath Sports Training Village gym, gym classes, and swimming pool, and a personalised feedback sheet of their own data presented in lay terms.

### Preliminary screening

During an initial telephone contact initiated by potential participants, a member of the study team will provide a verbal overview of the study, and ask if the participant believes that they meet all of the inclusion criteria and none of the exclusion criteria. Those who are interested in participating will be sent information about the study requirements in writing. Interested participants will be asked to sign an informed consent form stating their willingness to undergo an eligibility screening with a member of the research team to ensure that participants do not exhibit any physiological condition that either poses undue personal risk or introduces bias into the experiment. During this screening, participants will undertake an objective assessment of physical function (Short Physical Performance Battery (SPPB) [29]). Only participants scoring over 7 out of 12 without scoring 0 on any element of the SPPB will be eligible to participate. Balance scales and a stadiometer will be used to determine the participant’s weight and height, recorded on inhalation of breath with the head positioned in the Frankfort plane position. BMI will subsequently be calculated. Participants will then be provided with a pedometer (CW600 Digi-Walker, Yamax, Tokyo, Japan) and instructed in its use, a physical activity monitor BodyMedia armband (Sensewear Professional 8.0, Pittsburg, PA, USA) and dietary record sheets. Participants will be asked to wear the pedometer and the BodyMedia armband for 7 days and to weigh and record all food and drink consumed for 3 days (including one weekend day) using a diet record sheet. The pedometer will record the daily step count and the reset step count at midnight each day. Only individuals who average 3500 steps per day or above will be invited to take part in the rest of the study, and at this point one of the investigators will describe the study verbally to them again and provide the opportunity for potential participants to gain clarification on any aspect of the study. Eligible participants will be randomly allocated to an experimental group by minimisation to limit difference in mean age, steps/day and BMI at baseline between groups. All further details of the elements of the study specific to the group will be provided. Written informed consent will be acquired at this stage in regard to each participant’s understanding and willingness to take part in the rest of the study after group allocation. The investigator will also explain to the participants that they are completely free to withdraw from the study at any time without any repercussions.

### Familiarisation sessions

Participants will be asked to wear light clothes in which it is comfortable to perform exercise, and be given a thorough demonstration of all balance and strength tests, which they will then attempt.

Balance tests will assess postural sway with the participants’ eyes open and closed with them standing as still as possible on a force-measuring platform (AccuGait, AMTI, Watertown, MA, USA) with their feet side-by-side and heels 15 cm apart for 40 s, or feet together for 60 s. Participants will then practice the standard Y-balance test of single-leg balance [31], involving pushing their non-standing leg as far as they can forward, behind and to the right, and behind and to the left, whilst maintaining balance on the standing leg.

Leg strength assessments will be conducted on a pneumatic leg press machine (A420, Keiser®, Fresno, CA, USA), instrumented to measure the force applied to the foot pedals with the velocity the pedals moved at 400 Hz. Following a standardised warm-up, participants will complete one repetition maximum (1RM) tests on the leg press for single repetitions up to the greatest resistance at which one repetition can be completed, with self-selected rest and increments in force between repetitions. Determination of 1RM is commonly used in adult populations [32–34] and is considered safe [35], valid and reproducible [36] in older populations. Following a recovery period of at least 5 min, the participants will then practice the incremental power test on the leg press that will be used in the main trials. In this test, participants will complete approximately 10 discrete repetitions of the leg press exercise, with the movement in each repetition performed as quickly as possible. The resistance will increase in each successive repetition, starting with very low resistance up to approximately the previously achieved 1RM, with increasing recovery periods between repetitions. Participants will then be asked to repeat a similar familiarisation and 1RM test on a pneumatic knee extension machine.

Finally, participants’ ability to generate their maximum possible force (i.e. neural drive) will be examined by maximal voluntary isometric calf muscle contractions, with concomitant surface-evoked twitch stimulation to produce maximal stimulated force. The participant will be seated on a custom-built device, with the ankle dorsi-flexed to 10° and the knee braced at 90° against a strain gauge to measure the force of isometric contraction of the plantar-flexor muscles. Stimulating electrodes will be attached to the skin surface of the calf muscle region, with the cathode and anode placed on the upper and lower thirds of the triceps surae respectively. Stimulation will be delivered with a DS7 stimulator (Digitimer, Letchworth Garden City, UK). Initially, the force of a maximum voluntary plantar-flexor contraction will then be obtained, followed by a maximum evoked from single twitches at rest. Thereafter, the participant will make three repeated attempts to match the previously obtained maximum voluntary force with a pulse of electrical stimulation (of the same voltage previously identified to evoke maximal twitch force) passed across the calf muscle to maximally stimulate the muscle at the point of maximum force. The difference between the maximum voluntary force and maximum twitch-evoked force will indicate the participant’s ability to maximally stimulate their muscle [37].

### Main trials

**Fig. 2:**
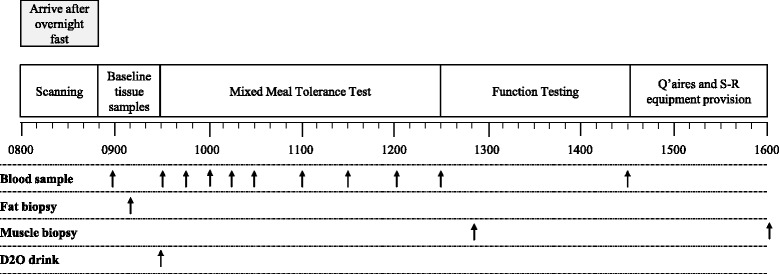
A schematic overview of the main trial data collection day; arrows indicate the timing of blood,adipose and muscle samples

The main trials will take place the day before the 14-day step-reduction (day 0) and the day after (day 15) (see Fig. 1). See Fig. 2 for schematic overview of the main trial day timeline.

Participants will be asked to report to the laboratory after a 10-hour overnight fast, wearing lightweight clothing with no metal components. Balance scales and a stadiometer will be used to determine the participant’s weight and height for calculation of BMI. A whole body dual-energy X-ray absorptiometry scan (DXA (Discovery; Hologic, Bedford, UK)) and a peripheral quantitative computed tomography scan ((pQCT) (XCT 3000 scanner; Stratec, Pforzheim, Germany)) of the calf (66 % tibia length) and thigh (25 and 50 % femur length) will be performed. An ultrasound scan of the medial gastrocnemius and vastus lateralis muscles will be conducted whilst standing to examine fascicle length and pennation angle (Echoblaster 128EXT-1Z, Revision C, and LV7.5/60/96z Transducer, LS128 CEXT, both TELEMED, Vilnius, Lithuania). The pQCT and ultrasound will be taken from the dominant leg. Subsequently, participants will complete five self-paced walks of 5 m whilst being filmed at 200 Hz with one camera in the sagittal plane to assess lower limb gait coordination.

A resting venous blood sample via cannula, followed by a biopsy sample of subcutaneous adipose tissue by needle aspiration, from approximately 5 cm lateral to the umbilicus, will be obtained after 15 min of seated/supine rest. Participants will then ingest a 200-ml dose of D_2_O water and undertake a mixed-meal tolerance test (mixed-macronutrient liquid meal of 2 g/kg BM carbohydrate; 0.8 g/kg BM fat; 0.4 g/kg BM protein) with 5-ml blood samples taken 15, 30, 45, 60, 90, 120, 150 and 180 min after consumption of the drink. Thereafter, a muscle biopsy will be sampled using the Bergstrom needle biopsy technique adapted for use with suction [38] from the vastus lateralis muscle. Consecutive muscle biopsies will be taken proximal to the previous biopsy site [39].

Participants will then undertake the balance testing as performed in the familiarisation procedure. The standard leg pressing warm-up will then be performed using the previously obtained 1RM, and the incremental power test as a measure of leg power will be undertaken. Following 2 min of recovery, participants will complete the assessment of neural drive of the calf muscle, followed by three sets of eight repetitions at 70 % 1RM on the leg press and leg extension (total of 48 repetitions). There will be 2 min of recovery between sets. Blood samples will be taken immediately on completion of exercise, and a muscle biopsy obtained from the contralateral leg after 90 min of rest. Whilst the participant rests, they will be asked to complete two questionnaires (The Pittsburgh Sleep Quality Index [40] and the World Health Organisation Quality of Life-BREF (WHOQOL-BREF) [41]).

### Step-reduction intervention

For 14 days participants will be asked to restrict their daily step count to 1500 steps per day. A portable pedometer will be worn by participants at all times throughout the day (excluding sleep) to allow accurate monitoring and feedback of step count. Step count will be recorded each day by the participant in a provided log-book. The pedometer will also automatically record daily step count, and will reset at midnight. An advice sheet will be provided to suggest strategies to reduce daily stepping. Participants will also wear a BodyMedia armband for the first 7 days of step-reduction. They will be asked to record their dietary intake on the first, seventh and 13th days of the step-reduction intervention using a dietary record sheet. For the two visits to the laboratory during the step-reduction period, transport arrangements will be made to minimise walking for the participant for the sake of the visits (i.e. door-to-door transport, transport within the facility/campus by wheelchair).

### Further assessments

On the third day of the 14-day step-reduction (day 3), participants will be asked to return to the laboratory for a blood sample and resting muscle biopsy. Participants in the exercise snacking group (ES-group) will be asked to perform an exercise snack in the laboratory. This will be repeated on the eighth day of the 14-day step-reduction (day 8), and 100 ml D_2_O water will be ingested.

Three days after the 14-day step-reduction (day 18), participants will be asked to return to the laboratory for a final resting blood sample and muscle biopsy to be taken.

### Exercise interventions

#### Pre-training group (PT)

The PT-group will attend 10 resistance training sessions across 4 weeks prior to the 14-day step-reduction, with sessions separated by at least 48 hours arranged at the participant’s convenience. The last training session will take place at least 3 but less than 7 days before the first main trial prior to step-reduction. All exercise sessions will be fully supervised by a member of the research team. Participants will complete the standardised warm-up on the Keiser leg press as performed during the familiarisation session. All training sessions will begin with leg press and leg extension, then alternate between two sessions (A and B) to incorporate variation into the programme (see Table 1). At the beginning of the fifth session, participants will repeat the 1RM and incremental power tests so exercise resistances can be adjusted to strength gains accordingly. Repetitions will be performed at a cadence of 1:1 for concentric-eccentric time, at a comfortable participant-selected pace that should be less than 5 s per complete repetition. Rest intervals between sets will be 90 to 120 s at the participant’s discretion.

**Table 1:** Training session content and progression

Exercise		Sessions 1 and 2			Session 3–10		
Session A	Session B	Sets	Reps	Load	Sets	Reps	Load
Seated leg press		3	8	60 % 1RM	4	8	75 % 1RM
Seated leg extension							
DB calf raise	DB lat raise	2	10	70 % 10RM	3	10	80 % 10RM
DB bicep curl and press	RB seated row						

*1RM* maximum load at which a single repetition can be completed, *10RM* maximum load at which 10 repetitions can be completed, *DB* dumbbells, *Lat* lateral plane of motion, *RB* elastic resistance band, *Reps* repetitions

#### Exercise snacking regime

The exercise snacking requires only a stable kitchen chair to perform it, and no supervision is necessary. Participants will be asked to perform five exercises, each for 1 min followed by 1 min of rest. In that minute of exercise, the participant will perform as many controlled repetitions of the given exercise as possible. Participants will be asked to perform the exercise snacks three times daily at least 2 hours apart. The exercises are: sit-to-stand from a chair, seated alternating knee extension, standing alternating knee bends holding onto a chair, marching on the spot, and standing calf raises. Participants will always perform the sit-to-stand exercise first, and record the number of repetitions completed within 1 min, and thereafter participants may alternate the order of exercises, but will be asked to record the order in a log-book provided. Participants will be asked to record in the log book the number of steps taken immediately prior to, and after, each exercise snacking bout so as to not count the steps incurred for each exercise snack towards the 1500 steps/day limit.

#### Re-training

Participants will attend six resistance training sessions after the step-reduction, with the first training session taking place on the day of follow-up biopsy 3 once the sample has been taken. The remaining five sessions will take place over 14 days with sessions separated by at least 48 hours. These sessions will follow the same training regime as the PT-group undertook prior to commencing the step-reduction. Resistances for the exercises during re-training will be based on the data from the main trial post step-reduction.

#### Post re-training muscle size, strength and power assessments

Follow-up assessments of muscle function, balance, and muscle size and architecture will be made 2 days after the final session of the 2-week laboratory-based re-training. This assessment will include repetition of the DXA, pQCT and ultrasound scans, and the leg press incremental power test, test of neural drive, and standing balance.

### Acceptability and feasibility of adopting prescribed training regimes

Following the 2-week re-training period participants will be invited to take part in an interview to provide feedback on their experience during the trial to inform the feasibility assessment of each exercise regime. Participants will be provided with written information of the purpose of the interviews following their final re-training session, and reassured that participation is optional. Interviews will take place via telephone or in person according to participant preference, will be digitally recorded, transcribed verbatim and anonymised on completion. The interview schedule will include: (1) participants’ perceptions of the impact of inactivity on mood, appetite, vitality, sleep and other participant-generated concerns (for comparison across conditions), and (2) perceived barriers and facilitators and costs and benefits to adherence to the study protocol (i.e. if and how participants adapted the prescribed regimes, and factors they suggest would make the protocol easier to adopt).

### Sample processing and storage

Adipose and muscle tissue samples will be processed and analysed immediately on the day of collection or stored at −80 °C for later batch analysis. Blood collected into an ethylenediaminetetraacetic acid (EDTA) tube (2 ml) will be used to provide a full leukocyte differential and other standard haematology variables with an automated haematology analyser (SF-300, Sysmex Ltd., Milton Keynes, UK). Other blood samples collected into EDTA and plain tubes (5 ml) will be centrifuged to obtain plasma and serum, respectively. Blood collected into a sodium heparin tube (25 ml) will be used to isolate peripheral blood mononuclear cells. All samples will be stored at −80 °C for later batch analysis.

### Potential harms

An inherent risk associated with this study is the potential for loss in lean tissue and muscle function for participants; however, the graduated exercise re-training programme will aim to fully restore decrements in muscle size and function. Moreover, all exercise sessions and testing will be supervised by study staff with appropriate training. The blood and tissue sampling techniques used in the present study also carry a risk of discomfort to the participant; however, all study staff are fully trained, experienced, and adhere to best practice at all times to minimise this risk. All tissue sampling procedures will be performed in sterile conditions, and participants will be provided with extensive advice on aftercare of biopsy sites to minimise risk of infection. Any adverse events will be recorded, assessed, and monitored by the University of Bath sponsorship in the form of 6-monthly progress reports. All potential risks have been reported to, assessed, and approved by the study sponsor, Research Ethics Approval Committee for Health (University of Bath), and the Cornwall and Plymouth NHS Research Ethics Committee. Any modifications to the protocol will be approved by all aforementioned parties.

### Data analyses

#### Primary outcome measure

Differences in leg power from pre- to post-step-reduction intervention and differences between groups.

#### Secondary outcome measures

Differences from pre to post step-reduction within and between groups in (1) leg muscle size, (2) muscle fascicle length and pennation angle, (3) body composition, (4) standing balance ability, (5) distances reached on the Y-balance test, (6) coordination variability in walking, (7) neural drive to muscle, (8) postprandial area under the curve of circulating insulin, triglyceride, and postprandial high-density lipoprotein (HDL), low-density lipoprotein (LDL) and non-esterified fatty acid (NEFA) concentrations, (9) rates of muscle protein and adipose triglyceride synthesis at rest over 14 days as assessed by D_2_O incorporation into muscle protein and glycerol in adipose, respectively, (10) total and phosphorylated muscle protein synthesis (protein kinase B (AKT), mechanistic target of rapamycin (mTOR), 70-kDa ribosomal protein S6 kinase 1 (P70S6K), 4E binding protein 1 (4EBP-1)) and breakdown (muscle RING-finger protein-1 (MuRF-1), muscle atrophy F-box (MAFbx), forkhead box (FOXO)) of signalling proteins within the muscle, (11) muscle heat shock protein (HSP)27 content before and after exercise, (12) subcutaneous adipose tissue cytokine secretion and gene expression (e.g. leptin, adiponectin, interleukin (IL)-6, tumour necrosis factor (TNF)α, IL-8, IL-1ra) (13) systemic cytokines at rest (e.g. IL-6, TNFα, C-reactive protein (CRP)), (14) mitochondrial function at rest (e.g. H_2_O_2_ and aconitase activity), and (15) T-lymphocyte interferon-γ production following stimulation with proteins from common viruses (e.g. adenovirus, influenza, *Varicella zoster* virus, cytomegalovirus, Epstein-Barr virus).

There is no intention to terminate the trial based on any interim results collected, and thus no data monitoring committee is deemed necessary. No ancillary data analysis is anticipated. Only study staff will have access to the trial dataset until all future manuscripts containing results are written, and only in anonymised format. Authorship eligibility will be addressed on completion of data analysis.

### Acceptability and feasibility

Interview transcripts will be analysed systematically using framework analysis to highlight key themes within and across participants relevant to the acceptability of the two intervention protocols, and the feasibility of wider implementation.

### Statistical analyses

Repeated measures analysis of variance (ANOVA) with appropriate post-hoc tests adjusted for multiple comparisons will examine changes over time in the key variables measured in muscle (e.g. HSP content, total muscle protein synthesis, phosphorylation of muscle protein, synthesis and breakdown of signalling proteins, etc.) and blood samples (e.g. inflammatory variables, biomarkers of metabolic health). Paired *t* tests will be employed to test for differences in body composition variables, strength and power, neural drive to muscle, balance, adipose tissue cytokine secretion and insulin sensitivity pre versus post interventions. Correlation coefficients will also be calculated between transcription factor activation and phosphorylation of signalling proteins. Statistical significance will be accepted at *P* <0.05. Effect sizes will also be calculated according to Cohen [42], with *d* >0.08 considered a large effect. Magnitude-based inferences will also be calculated to examine the effect of the exercise strategies on all variables of interest.

## Discussion

This study aims to evaluate the effectiveness of two exercise strategies to attenuate the effects of 2 weeks of reduced activity on muscle power and other key health indices that are known to be affected by changes in physical activity level. Based on the previous studies, specifically implementing a daily step-reduction in older individuals [15, 21], it is expected that 14 days of reduced physical activity without protective interventions will have a significant physiological effect, with reductions in both muscle mass and insulin sensitivity. These changes, however, are reversible and participants will undergo re-training and assessments of muscle size and function will be made to ensure participants have returned to baseline (i.e. pre step-reduction). The step-reduction model and the exercise interventions incorporated into this study have been selected specifically because of their real-world applicability and high ecological validity. It is hoped that the exercise interventions will provide a proof of principle that even relatively modest exercise interventions may be effective countermeasures against reduced activity-induced muscle atrophy and decline in other health indices, and that these intervention can be further developed and optimised in future work. Given the limited previous literature using a reduced-activity model in older individuals it is hoped that inclusion of qualitative feasibility analysis may draw out questions of interest that may not have been previously considered, as well as considerations to improve future work in this field.

### Study limitations

The findings of the present study will be inherently limited in applicability due to the inclusion of only healthy, non-smoking, non-obese, non-frail men, and the exclusion of more common clinical phenotypes for this age group. Moreover, the findings will hold limited generalisability to older individuals taking regular anti-inflammatory medication and to older women. A further limitation is that this study will not be able to inform on recovery from reduced activity when simply returning to habitual activity levels due to inclusion of the exercise re-training for all groups. It is also not possible to draw conclusions on the efficacy of the pre- and re-training programmes in altering any markers of health measured from tissue or blood samples, some of which may provide evidence as the mechanisms of change at the whole body level.

### Trial status

Participant recruitment for this study is ongoing.

## Abbreviations

1RM, one repetition maximum; 10RM, 10 repetitions maximum; 4EBP-1, 4E binding protein 1; AKT, protein kinase B; ANOVA, analysis of variance; BMI, body mass index; CRP, C-reactive protein; D_2_O, deuterium oxide-labelled water; DXA, dual-energy X-ray absorptiometry; EDTA, ethylenediaminetetraacetic acid; ES-group, exercise snacking group; FOXO, forkhead box; H_2_O_2_, hydrogen peroxide; HDL, high-density lipoprotein; HSP, heat shock protein; IL, interleukin; LDL, low-density lipoprotein; MAFbx, muscle atrophy F-box; mTOR, mechanistic target of rapamycin; MuRF-1, muscle RING-finger protein-1; NEFA, non-esterified fatty acid; P70S6K, 70-kDa ribosomal protein S6 kinase 1; pQCT, peripheral quantitative computed tomography; PT-group, pre-training group; SPPB, Short Physical Performance Battery; TNFα, tumour necrosis factor alpha; WHOQOL-BREF, World Health Organisation Quality of Life-BREF questionnaire.
